# Effects of silver nanoparticles on *Staphylococcus aureus* contaminated open wounds healing in mice: An experimental study

**Published:** 2017-03-15

**Authors:** Masood Adibhesami, Malahat Ahmadi, Amir Abbas Farshid, Farshid Sarrafzadeh-Rezaei, Bahram Dalir-Naghadeh

**Affiliations:** 1 *PhD candidate, Department of Microbiology; Faculty of Veterinary Medicine, Urmia University, Urmia, Iran.*; 2 *Department of Microbiology; Faculty of Veterinary Medicine, Urmia University, Urmia, Iran.*; 3 *Department of Pathobiology; Faculty of Veterinary Medicine, Urmia University, Urmia, Iran.*; 4 *Department of Surgery and Diagnostic Imaging; Faculty of Veterinary Medicine, Urmia University, Urmia, Iran.*; 5 *Department of Internal Medicine and Clinical Pathology, Faculty of Veterinary Medicine, Urmia University, Urmia, Iran.*

**Keywords:** Infection, Nanoparticle, Silver, Skin, Wound healing

## Abstract

The microorganisms have been noted as the main cause of delayed wound healing. The most common pathogen causing the wound infections is *Staphylococcus aureus*. Silver nanoparticles (AgNPs) show ample antibacterial activities. In the present study, the effect of AgNPs on mouse wounds inoculated with *S. aureus* was investigated. Sixty male mice (20 to 30 g) were anesthetized, full-thickness skin wounds were made on their back and then the bacterial suspension was added to each wound bed. Treatments were administered on wound bed topically including gentamicin (8 mg kg^-1^), AgNPs (0.08 mg kg^-1^, 0.04 mg kg^-1^ and 0.02 mg kg^-1^) and normal saline in the control group. Wound healing was monitored macroscopically by taking digital photographs on days 0, 7, 14 and 21 of the experiment. Topical application of gentamicin and AgNPs (0.08 and 0.04 mg kg^-1^) significantly increased the rate of wound healing more than treatment with AgNPs at a dose of 0.02 mg kg^-1^and normal saline. The presence of silver nanoparticles in AgNPs groups (especially 0.08 mg kg^-1^) improved wound appearance better than other groups without silver nanoparticles (gentamicin and control groups) and led to lesser wound scars. According to data analysis, healing rate of treated mice with gentamicin and AgNPs (0.08 mg kg^-1^) was significantly (*p *< 0.001) faster than treated mice with other AgNPs doses and normal saline. The results of current study introduced an *in vivo* nanosilver accelerating effects on the treatment of on *S. aureus* infected skin wounds.

## Introduction

One of the main objectives in wound healing is restoration in the shortest time with minimal side effects.^1^ Today, infection is considered as a first cause of mortality owing to wounds, especially after surgery.^2^ *Staphylococcus aureus* has an important role in the infection after surgery from ancient times.^3^ In this regard, several antimicrobials agents were used, but each one had serious limitations or side effects such as delaying of wound healing and bacterial resistance to antibiotics.^4^ Therefore, less expensive methods with fewer side effects have to be applied.

Nanotechnology puts together the capabilities to manage the properties of materials by controlling their size and this has motivated carrying out the researches into numerous potential uses for nanomaterials.^5^ An ideal material should be able to protect wounds against microbial interactions.^6^ For centuries, metals including silver, gold and zinc have been applied as bactericidal and bacteriostatic agents, each with different properties and spectrums of activity. Antimicrobial properties of silver nanoparticles have been proven and they were used, for instance, in food industry, hygiene materials and detergents.^7^^,^^8^ Also, minimum inhibitory concentration of silver nanoparticles is lower than that of silver ions.^9^

There is an increase in the medical application of nanoparticles in the process of wound healing.^10^ In the present study, we have investigated the effect of silver nanoparticles (AgNPs; 20 nm in diameter) on surgically induced full thickness skin wounds inoculated with *S. aureus* and superficial and deep bacterial load of wounds’ local infection in mice.

## Materials and Methods

All procedures in this study were carried out in accordance with the guidelines of the Animal Ethics Committee of Faculty of Veterinary Medicine, Urmia University (AECVU) and supervised by authority of Urmia University Research Council (UURC). 

**Animals. **Adult male albino mice of seven weeks old (20 to 30 g) were used in this study. Mice were divided into five groups (n = 12) randomly, kept under periods of 12 hr light and 12 hr dark, specific pathogenic-free conditions. The mice were kept on standard pellet diet and water *ad libitum* for two weeks to be acclimatized prior to the investigation.^11^

**Preparation of bacteria. **The *S. aureus* (ATCC 25923) was obtained from MAST Company Oswestry, UK. The bacteria were cultured in Muller-Hinton broth (Merck, Darmstadt, Germany) and in the log phase of growth, the suspension was centrifuged at 1000 g for 15 min. The supernatant was discarded and the bacteria were diluted to 10^8^ CFU mL^-1^ in sterile phosphate-buffered saline. Ten µL of the bacterial suspension (10^6^ CFU) were added to each wound bed after induction of full thickness skin defect, immediately.^12^

**Creation of full thickness skin defect. **All mice were anesthetized with intra-peritoneal injection of 250 µL ketamine-xylazine-saline cocktail (ratio 4:1:35) consisting of 100 mg kg^-1 ^ketamine (Alfasan, Woerden, Holland) and 5 mg kg^-1 ^xylazine (Alfasan).^13^ The skin area of the back of mice were prepared aseptically for surgery and full thickness skin wounds (4 mm in diameter) were made on dorsal midline using sterile biopsy punch equipment (Revolving punch pliers, Dimeda, Württemberg, Germany). The wounds were left open without any dressing during the study.^14^ As mentioned before, the mice were randomly divided into five groups of 12 animals including control (normal saline), gentamicin (8 mg kg^-1^; Caspiantamin, Tehran, Iran), AgNPs (0.08 mg kg^-1^), AgNPs (0.04 mg kg^-1^) and AgNPs (0.02 mg kg^-1^). In all groups, treatments were applied in the wound bed topically only once on day 0.^15^

**Wound infection measurement. **This was measured using evaluation of bacterial load at the wound site on days 7, 14 and 21 applying two methods. First, a swab test was executed from the wound surface for analyzing surface bacteria on days 7 and 14. The sample was then carried to a proper diluent. In order to assessment of bacterial concentration, the suspensions were serially diluted from 1:10^3^ to 1:10^12^ with sterile broth media (Merck) and each dilution was placed on broth-agar plates. The second test assessed infection of tissue depth on the 21^st^ day. The skin was excised including the entire wound with the nearby normal skin. The tissue biopsies were homogenized in a tissue grinder after weighing and then homogenized in 1.50 mL of Muller-Hinton Broth (Merck). For hypothetical evaluation, a drop of the homogenate was placed on the slide and stained using Gram method (the presence of one or more micro-organisms per oil-immersion field reflects a microbial load at least of 10^5^ CFU g^-1^).^16^ For serial dilutions preparation, the homogeneous tissue 1:10 (0.10 + 0.90) was placed in the dilution blanks using normal saline. The CFU per gram of tissue was calculated by the following equation:^17^

*CFU g* ^-1^ *= [Plate count × (1 per dilution) × 10] / Weight of homogenized tissue*

**Wound size measurement. **All wounds were photo-graphed at deﬁned time points (all photos were taken from a distance of 25 cm of wound sites with eye level shot angle without zooming). Day 0 photographs were taken immediately after the injury. The wound healing was investigated by capturing digital photographs on days 0, 7, 14 and 21 of the experiment. In order to evaluate healing performance, the induced wounds scar sizes were measured using Adobe Photoshop histogram analysis (version 10; Adobe System Incorporated, San Jose, USA). Wound size which states the quantity of the wound area, was determined on every post-treating day and compared with the initial wound area using the below formula: ^18^

*wound contraction (%)* *=* *[(A*_0_ *– A*_t_ *)/A*_0_ *] × 100*

where, *A*_0_ is the initial wound area and *A*_t_ is the area of wound at the time of image capturing and bacterial counting (on days 0, 7, 14 and 21, respectively). Areas were evaluated by the images of the wounds using image analysis software after calibration in same days. The animals were euthanized with CO_2_ inhalation.

**Statistical analysis. **Statistical analyses of data were done using the SAS software (version 9.4; SAS Institute Inc., Cary, USA). Comparison of the effects of AgNPs, gentamicin and normal saline in different groups and time on wound area, diameter and contraction were done by mixed methods (proc mixed) and repeated expressions. Preliminary data assessment using the box plots was performed for the presence of outliers, homogeneity of variance and data distribution. To estimate assumptions, square root and/or natural log data were analyzed. Constant variables of model were included group, time and the interaction between them. Mice were random effects of model. The area and diameter in day 0 were used as the covariate data. Various covariance structures (first-order auto-regressive, heterogeneous first-order autoregressive, first-order ante-dependence, Toeplitz, unstructured, compound symmetry, and heterogeneous compound symmetry) for choosing a suitable model of solidarity and variance during time were evaluated according to the Akaike’s information criterion and the best structure selected on the basis of the lowest criteria mentioned. Survey of residuals against predicted values was used to assess the final model. Compare of groups’ pair was performed using Bonferoni test. The results of these variables are shown as a least-square means ± SEM least squares means.

## Results

**Fig. 1:**
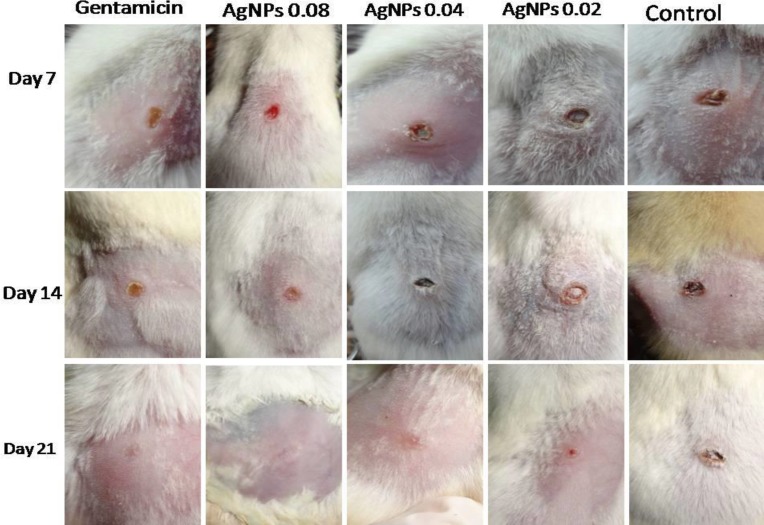
Effects of topical application of gentamicin(8 mg kg^-1^), AgNPs (0.08 mg kg^-1^), AgNPs (0.04 mg kg^-1^) and AgNPs (0.02 mg kg^-1^) on the healing of full-thickness wounds in mice. Representative images of mice from groups taken on 7, 14 and 21 days after creation of wound are shown

Among the animals, healing rate of treated mice with gentamicin (8 mg kg^-1^), AgNPs (0.08 mg kg^-1^) and AgNPs (0.04 mg kg^-1^) was significantly (*p *< 0.001) faster than mice treated with AgNPs (0.02 mg kg^-1^) and untreated ones from days 7 to 14, but no differences were observed between control group and AgNPs (0.02 mg kg^-1^) and between gentamicin (8 mg kg^-1^) and AgNPs (0.08 mg kg^-1^) in day 7. The results showed that there are significant differences (*p *< 0.001) between control group and AgNPs (0.08 mg kg^-1^), AgNPs (0.04 mg kg^-1^) and AgNPs (0.02 mg kg^-1^), and gentamicin (8 mg kg^-1^) and AgNPs (0.02 mg kg^-1^) in day 14. Finally, no difference was observed among all groups in day 21 (Fig. 1).

After treatment with gentamicin (8 mg kg^-1^) and AgNPs (0.02, 0.04, 0.08 mg kg^-1^), the wound bed areas decreased significantly (*p *< 0.0001) from day 7 to day 21. There was significant difference (*p *< 0.0001) between AgNPs (0.08 mg kg^-1^) group with other groups and gentamicin with control group at day 7. No significant differences observed between control group and AgNPs (0.02 mg kg^-1^) and gentamicin (8 mg kg^-1^) group with AgNPs (0.08 mg kg^-1^) in day 14 (Table 1). 

**Table. 1:** Effect of topical application of gentamicin (8 mg kg^-1^), AgNPs (0.08 mg kg^-1^), AgNPs (0.04 mg kg^-1^) and AgNPs (0.02 mg kg^-1^) on wound area (mm^2^). Data are presented as mean ± SEM

**Groups**	**Wound area (%wound contraction )**			
	**Day 0**	**Day 7**	**Day 14**	**Day 21**
**Control**	12.56	11.05 ± 0.15 (11.39)	6.43 ± 0.18 (48.59)	1.54 ± 0.25 (87.11)
**Gentamicin**	12.56	9.66 ± 0.15 (23.00)	2.67 ± 0.18 (78.94)	0.00 ± 0.25 (99.86)
**AgNPs (0.08 mg kg** ^-1^ **) **	12.56	8.57 ± 0.15 (31.81)*	3.20 ± 0.19 (74.75)	0.11 ± 0.26 (99.93)*
**AgNPs (0.04 mg kg** ^-1^ **)**	12.56	9.97 ± 0.15 (20.52)	4.57 ± 0.18 (64.29)	0.13 ± 0.25 (98.70)
**AgNps (0.02 mg kg** ^-1^ **)**	12.56	10.50 ± 0.15 (16.28)	5.66 ± 0.18 (53.78)	1.22 ± 0.25 (89.92)

* The percentage of wound was contraction compared with day 0 is provided within the parentheses. Asterisks denote *p-value* signiﬁcance comparing AgNPs 0.08 mg kg^-1^ to the other groups (*p *< 0.0001).

Topical application of gentamicin, AgNPs (0.08 mg kg^-1^) and AgNPs (0.04 mg kg^-1^) increased the rate of wound healing significantly more than AgNPs (0.02 mg kg^-1^) and normal saline group. Wound healing in the genatmicin, AgNPs (0.08 mg kg^-1^) and AgNPs (0.04 mg kg^-1^) groups were nearly complete in day 21 after wound induction, whilst the wound in mice of AgNPs (0.02 mg kg^-1^) and control groups had not been healed entirely (Table 1). No side effects were observed on body weight, general health and behavior of animals during treatment.

Increased wound closure was observed in groups treating with AgNPs and gentamicin at days 7 and 14, respectively. Almost all wounds in animals except control and AgNPs (0.02 mg kg^-1^) groups’ ones reached 100% closure at day 21.

Data analysis showed that good inhibition of bacterial growth (*S. aureus*) was gained in all groups treated with AgNPs or gentamicin. *Staphylococcus aureus* count in control group was significantly higher than treated mice and was more than 300 colonies on medium (defined as uncountable) in first two weeks and some wounds appeared infected, obviously (Fig. 1). A significant reduction in wound bacterial count was obtained in treated groups. The AgNPs (0.04 mg kg^-1 ^and 0.02 mg kg^-1^) groups had a slight but significant reduction in bacterial count compared to control group, reaching to 1 CFU g^-1^ (total count) and 0 CFU g^-1^ (*S. aureus* count) in day 21. The highest inhibition of bacterial growth was obtained in groups receiving AgNPs (0.08 mg kg^-1^) and gentamicin (Table 2). 

**Table 2:** Bacterial count of wound bed in treated and untreated mice

**Organism**	**Groups**	**Day**	**Bacterial count (CFU 10 µL** ^-1^ **)**
**Total**	**Control**	0 7 0 14 0 21	5.22 × 10^4^ Uncountable 5.52 × 10^7^ Uncountable 5.42 × 10^4^ 83.00 CFU g^-1^
***S. aureus***		0 7 0 14 0 21	5.52 × 10^4^ Uncountable 5.28 × 10^4^ 7.21 × 10^5^ 5.63 × 10^4^ 12.00 CFU g^-1^
**Total**	**AgNPs ** **(0.08 mg kg** ^-1^ **)**	0 7 0 14 0 21	5.28 × 10^4^ Uncountable 5.38 × 10^4^ 1.75 × 10^4^ 5.48 × 10^4^ 0.00 CFU g^-1^
***S. aureus***		0 7 0 14 0 21	5.28 × 10^4^ Uncountable 5.28 × 10^4^ 1.02 × 10^4^ 5.28 × 10^4^ 0.00 CFU g^-1^
**Total**	**Gentamicin**	0 7 0 14 0 21	5.41 × 10^4^ Uncountable 5.28 × 10^4^ 2.37 × 10^4^ 5.28 × 10^4^ 0.00 CFU g^-1^
***S. aureus***		0 7 0 14 0 21	5.31 × 10^4^ Uncountable 5.27 × 10^4^ 3.24 × 10^4^ 5.29 × 10^4^ 0.00 CFU g^-1^
**Total**	**AgNPs ** **(0.04 mg kg** ^-1^ **)**	0 7 0 14 0 21	5.29 × 10^4^ Uncountable 5.28 × 10^4^ 2.65 × 10^4^ 5.26 × 10^4^ 1.00 CFU g^-1^
***S. aureus***		0 7 0 14 0 21	5.33 × 10^4^ Uncountable 5.28 × 10^4^ 2.01 × 10^4^ 5.25 × 10^4^ 0.00 CFU g^-1^
**Total**	**AgNPs ** **(0.02 mg kg** ^-1^ **)**	0 7 0 14 0 21	5.28 × 10^4^ Uncountable 5.23 × 10^4^ 2.89 × 10^4^ 5.29 × 10^4^ 1.00 CFU g^-1^
***S. aureus***		0 7 0 14 0 21	5.33 × 10^4^ Uncountable 5.24 × 10^4^ 2.01 × 10^4^ 5.28 × 10^4^ 0.00 CFU g^-1^

## Discussion

Nanomaterials with antimicrobial activity that elevate the effectiveness and safety of antimicrobial administration are called nano-antibiotics. ^19^ Their capability in control of infection has been explored and demonstrated *in vitro* and *in vivo*.^20^ Due to prompt prevalence of multidrug-resistant pathogens and insufficient research regarding antibiotic production; the AgNPs could be useful alternatives of routine antibiotic therapy.

Attention to the wound tissue remodeling and its infection is critical for quick repair with no side effect. Wound healing is a treatment priority especially for diabetic or infected wounds suffering patients.^21^

Silver is a broad-spectrum antimicrobial that inhibits growth of microbes.^22^ It has been previously shown that AgNPs have *in vitro* antibacterial activities against *S. aureus*.^23^ In this study, we report *in vivo* capabilities of AgNPs that appear to accelerate healing of wounds inoculated with *S. aureus* in a mouse model of skin wound. This study showed that AgNPs dilutions accelerate wound healing and this acceleration is more noticeable in 0.08 mg kg^-1^ level. Reportedly, AgNPs are responsible for reducing the time required for hyperactive cells (myofibroblasts) involved in generation of contractile force in the wound and reverse the inflammatory processes more quickly compared to antibiotic application.^24^^,^^25^ Topical application of AgNPs effectively enhanced the remodeling of wounds area and diameter and skin macroscopic appearance in mice. In this study, it was observed that 14 and 21 days after treatment, in groups treated with gentamicin or different dilutions of AgNPs, total and *S. aureus* bacterial loads reduced significantly. Gentamicin and AgNPs (0.08 mg kg^-1^) were more effective than AgNPs (0.04 mg kg^-1^) and AgNPs (0.02 mg kg^-1^) in reducing bacterial loads, so total and *S. aureus* amounts were 0 CFU g^-1^ in day 21 after wound induction. In control group, 7 and 14 days after wound induction, the amounts of total bacteria and 7 days after wound induction, the amounts of *S. aureus* increased, so they were uncountable in plate. This difference in total and *S. aureus* amounts may be due to antibacterial activities of AgNPs against gram-negative and gram-positive bacteria. There is no clinical study about the effects of AgNPs on bacterial load, however, the *in vitro* antibacterial effects of AgNPs are well documented.

Antimicrobial mechanisms of nanomaterials include photocatalytic production of reactive oxygen species that damage cells, compromising the bacterial cell wall or membrane, interruption of energy transduction and inhibition of enzyme activity and DNA synthesis.^20^^,^^26^

Our findings support the potent antibacterial activities of AgNPs as antibacterial agents in infected wounds and suggest their use in preparing medical devices such as indwelling vascular catheters. These devices can prevent the proliferation and colonization of opportunistic bacteria in patients.

The results of this study introduced a remarkable *in vivo* nanosilver accelerating effects on the treatment of on *S. aureus* infected skin wounds with no obvious side effects in mice. 

## References

[1] Rizzi SC, Upton Z, Bott K (2010). Recent advances in dermal wound healing: Biomedical device approaches. Exp Rev Med Dev.

[2] Burke JP (2003). Infection control - a problem for patient safety. New Eng J Med.

[3] Bratzler DW, Houck PM (2004). Antimicrobial prophylaxis for surgery: An advisory statement from the national surgical infection prevention project. Clin Infect Dis.

[4] Liesbet DB, Coessens G, Boelens J (2013). Microbial etiology and antimicrobial resistance in healthcare-associated versus community-acquired and hospital-acquired bloodstream infection in a tertiary care hospital. Diagnostic Microb Infec Dise.

[5] Tian J, Wong KK, Ho CM (2007). Topical delivery of silver nanoparticles promotes wound healing. Chem Med Chem.

[6] Lansdown AB (2006). Silver in health care: ntimicrobial effects and safety in use. Curr Prob Derm.

[7] Lkhagvajava N, Yashab I, Chelik E (2011). Antimicrobial activity of colloidal silver nanoparticles prepared by sol-gel method. Dig J Nanomater Biostruct.

[8] Beveridge TJ, Phadtare MN, Lee H (1997). Adv Microb Physiol.

[9] Huh AJ, Kwon YJ (2011). Nanoantibiotics: A new paradigm for treating infectious disease using nanomaterials in the antibiotics resistant era. J Control Release.

[10] Ziv-Polat O, Topaz M, Brosh T (2010). Enhancement of incisional wound healing by thrombin conjugated iron oxide nanoparticles. Biomat.

[11] Baumans V, Van Loo PLP (2013). How to improve housing conditions of laboratory animals: The possibilities of environmental refinement. Vet J.

[12] Tanideh N, Rokhsari P, Mehrabani D (2014). The healing effect of licorice on Pseudomonas aeruginosa infected burn wounds in experimental rat model. World J Plast Surg.

[13] Tymen SD, Rojas IG, Zhou X (2013). Restraint stress alters neutrophil and macrophage phenotypes during wound healing. Brain Behav Immun.

[14] Michael LC, Xianfeng CHH, Mark AF (2013). Elastic modulus and viscoelastic properties of full thickness skin characterised at micro scales. Biomaterials.

[15] Yates CC, Whaley D, Babu R (2007). The effect of multifunctional polymer-based gels on wound healing in full thickness bacteria-contaminated mouse skin wound models. Biomaterials.

[16] Williams RL, Sroussi HY, Abercrombie JJ (2012). Synthetic decapeptide reduces bacterial load and accelerates healing in the wounds of restraint-stressed mice. Brain Behav Immun.

[17] Weir E, Lawlor A, Whelan A (2008). The use of nanoparticles in anti-microbial materials and their characterization. The Analyst.

[18] Jiang B, Larson JC, Drapala PW (2012). Investigation of lysine acrylate containing poly(N-isopropylacrylamide) hydrogels as wound dressings in normal and infected wounds. J Bio Mat Res.

[19] Etheridge ML, Campbell SA, Erdman AG (2013). The big picture on nanomedicine: The state of investigational and approved nanomedicine products. Nanomed.

[20] Pal S, Tak YK, Song JM (2007). Does the antibacterial activity of silver nanoparticles depend on the shape of the nano-particle? A study of the Gram-negative bacterium Escherichiacoli. Appl Enviro Microb.

[21] Mohammadi R, Rabbanib SH, Bahramic H (2016). Antibacterial performance and in vivo diabetic wound healing of curcumin loaded gum tragacanth/poly(ε-caprolactone) electrospun nanofibers. Mat Sci Eng.

[22] Anand KKH, Mandal BK (2015). Activity study of biogenic spherical silver nanoparticles towards microbes and oxidants. Spectrochim Acta A Mol Biomol Spectrosc.

[23] Kazemi J, Ahmadi M, Dastmalchi Saei H (2014). Antibacterial effect of silver nanoparticles along with protein synthesis-inhibiting antibiotics on Staphylo-coccus aureus isolated from cattle mastitis. Bio J Micro.

[24] Karim RZ, Adnan R, Ansari MS (2012). Low concentration of silver nanoparticles not only enhances the activity of horseradish peroxidase but alter the structure also. PLoS ONE.

[25] Lu PY, Lee CM (2010). Silver nanoparticles mediate differential responses in keratinocytes and fibroblasts during skin wound healing. Chem Med Chem.

[26] Kvitek L, Panacek A, Prucek R (2011). Antibacterial activity and toxicity of silver-nano silver versus ionic silver. J Phys Conf Ser.

